# Non-fusion procedure using PEEK rod systems for lumbar degenerative diseases: clinical experience with a 2-year follow-up

**DOI:** 10.1186/s12891-016-0913-2

**Published:** 2016-02-01

**Authors:** Weimin Huang, Zhengqi Chang, Ruoxian Song, Ke Zhou, Xiuchun Yu

**Affiliations:** Department of Orthopedics, General Hospital of Jinan Military Commanding Region, NO. 25 Shifan Road, Jinan, Shandong 250031 People’s Republic of China

**Keywords:** PEEK rod systems, Non-fusion procedure, Lumbar degenerative diseases, Pedicle-based dynamic stabilization

## Abstract

**Background:**

Polyetheretherketone (PEEK) rod system is a novel pedicle-based dynamic stabilization system. This study evaluated clinical and radiographic outcomes of non-fusion surgery by PEEK rod systems for treatment of degenerative lumbar diseases with a 2-year follow-up.

**Methods:**

From February 2012 to October 2012, 38 patients who underwent non-fusion surgery using PEEK rod systems were included in the study. Data on Oswestry Disability Index (ODI) score and Japanese Orthopaedics Association (JOA) score were collected and radiographs were obtained to evaluate disc height index (DHI) and range of motion (ROM) at each interval.

**Results:**

Both JOA and ODI scores significantly improved postoperatively. DHI showed a slight increase immediately after the surgery but gradually dropped below preoperative levels. Mean ROM values changed from 8.8° preoperatively to 1.8° at the 2-year follow-up point. Screw loosening occurred in one case at the 2-year follow-up.

**Conclusions:**

The preliminary results indicated a significant improvement in clinical outcomes and advantageous implant safety. The non-fusion procedure using PEEK rod systems might be a viable alternative for treatment of lumbar degenerative diseases. The distraction technique needs to be improved for better postoperative DHI.

## Background

Lumbar degenerative diseases such as degenerative disc herniation, lumbar spinal stenosis, and lumbar instability syndrome exert a substantial impact on daily life and functional capacity. Traditionally, decompression combined with fusion has been widely accepted as conventional surgical treatment for these diseases [1]. The application of intervertebral cages aided by pedicle-based stabilization with rigid titanium rods have promoted fusion rate and the volume of spinal fusion has increased at a high rate [2]. Despite the widespread use of lumbar spinal fusion operations, concerns exist regarding their clinical outcomes and complications. Increased stiffness at the instrumented level, pseudarthrosis, implant failure, and accelerated adjacent level degeneration have been documented postoperatively [3, 4].

To eliminate or minimize these adverse outcomes, less rigid constructs have recently been introduced for the treatment of lumbar degenerative diseases [5, 6]. These systems are defined as dynamic or semi-rigid stabilization systems. Dynamic stabilization systems such as Dynesys, FlexPLUS and ISObar TTL are designed to stabilize the abnormal segments, unload the stress on the lumbar discs and maintain physiological intervertebral motion, while semi-rigid stabilization systems such as polyetheretherketone (PEEK) rods and ostaPek are designed to improve load-sharing, promote fusion rate, reduce interface stress of implants and meanwhile maintain segmental balance [7].

PEEK rod systems were initially introduced for pedicle screw instrumentation and approved by the Food and Drug Administration in 2007. The PEEK material exhibits extraordinary thermostability, resistance to chemical and radiation damage, full biocompatibility and minimal toxicity in vivo [8, 9]. And above all, it has an elastic modulus between that of the cortical and cancellous bone [8, 10, 11]. Compared with the titanium rod (114 GPa), the less rigid PEEK rod (3.2 GPa) may alter load-bearing and control abnormal motion, which in consequence may promote the intervertebral bone fusion rate according to Wolff’s law [10–12]. Several clinical studies concerning the use of PEEK rod systems for fusion procedure are available in the published literature [13–16]. However, due to their small sample size, short follow-up and conflicting results, clinical outcomes are still controversial.

As mentioned above, PEEK rod systems are always defined as instruments for semi-rigid fixation. In fact, the concepts of dynamic and semi-rigid stabilization are not absolutely distinct. A similar biomechanical effect between PEEK rod systems and Dynesys was reported earlier [5]. To the best of our knowledge, Dynesys is designed to stabilize the treated segment without fusion [6, 17]. Therefore, theoretically PEEK rod systems might also be effective if used in non-fusion operations. In contrast to the large numbers of clinical studies on Dynesys, no clinical examination with special focus on the non-fusion procedure of PEEK rod systems is available to the best of our knowledge. We hypothesized that the non-fusion procedure using PEEK rod systems was also an effective way for addressing lumbar degenerative diseases, and conducted the current investigation to examine the reliability and validity of this assumption by evaluating clinical and radiographic outcomes in 38 consecutive cases.

## Methods

### Study design

Thirty-eight consecutive patients, who had undergone non-fusion fixation of the lumbar spine with the PEEK rod systems from February 2012 to October 2012, participated in this study. The research was approved by the Ethics Committee of the General Hospital of Jinan Military Commanding Region (No.201110) and all participants provided written informed consent. Clinical and radiographic data were prospectively collected. All cases were presented with symptomatic degenerative lumbar disease such as disc disease, stenosis, or instability and the conservative treatment with analgesics, physiotherapy and manipulation for at least 3 months had failed. The criteria for performing dorsal non-fusion stabilization were: lumbar spinal stenosis, lumbar instability syndrome, or lumbar disc herniation associated with evidence of spinal instability, and chronic low-back pain [18]. Spondylolisthesis with Meyerding grade II–IV, scoliosis with Cobb angle >10°, pathologic fractures of the vertebrae, severe osteoporosis with T-score <2.5, body mass index (BMI) > 40 kg/m^2^, presence of active infections, spinal metastases or ankylosing spondylitis were excluded from this examination.

Preoperative evaluation included standard anterior- posterior, lateral and flexion–extension lumbar spine fluoroscopy, CT and MRI scans. For patients aged over 50 years, a dual energy X-ray absorptiometry (DEXA) scan was conducted to determine the T-score.

### Surgical techniques

All surgeries were performed by the same team. After general anesthesia, the patient was placed in a prone position and a posterior midline approach was used to access the affected lumbar levels. Facet joints were exposed appropriately with careful preservation of the capsules. Pedicle screws were inserted transpedicularly and their correct positions were confirmed by C-arm fluoroscopy. Decompression of the involved nerve roots was performed. When necessary, it was completed by discectomy. After adequate decompression, proper-sized alloy rods were inserted and distraction was accomplished. Then similar-sized PEEK rods were positioned and connected to the pedicle screws in place of the alloy rods. Bone grafting was not used. Finally, suction drains were placed and the surgical wound was closed in layers. Patients received intravenous antibiotics for 24–48 h if necessary and were allowed to get up on the second postoperative day. All patients were requested to wear a lumbar brace for 12 weeks.

### Clinical effects evaluation

The clinical data for all patients were recorded preoperatively and postoperatively at five time points distributed at gradually increasing intervals: 1 week, 3, 6, 12 and 24 months. Clinical outcomes were quantified by Oswestry Disability Index (ODI) score and Japanese Orthopaedics Association (JOA) score.

### Radiologic outcomes evaluation

**Fig. 1:**
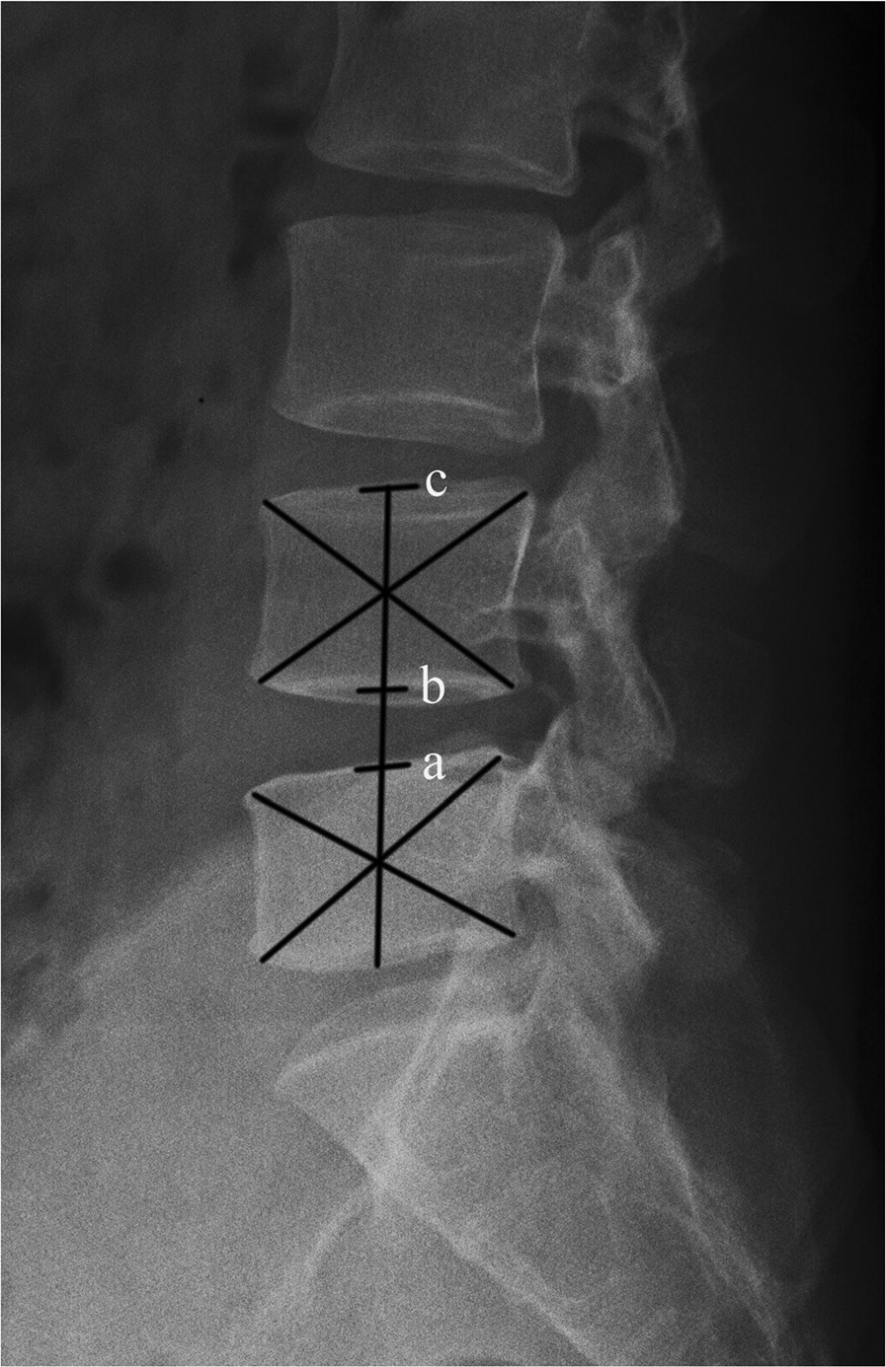
DHI (disc height index) measurement by *ab*/*bc. Ab* represents the disc height and *bc* represents the vertebrae height. *Ac* is the line labeling the centers. The center of the vertebral body is marked by the crossing point of two *diagonal lines*, which are drawn from the corners of vertebral body

Anterior-posterior and lateral lumbar radiographs were obtained at each interval. Disc height index (DHI) was calculated using the lateral view according to Kim’s method (Fig. 1) [19]. Screw loosening was defined as the presence of a “halo zone sign” or “double halo sign” on anterior-posterior radiograph during follow-up [20].

Flexion and extension X-rays were performed preoperatively and postoperatively at 3, 6, 12 and 24 months postoperatively. The segmental range of motion (ROM) was calculated as the difference between the segmental angulation in flexion and extension.

CT scan and three-dimensional reconstruction was accomplished at the final follow-up to confirm the integrity of PEEK rods. The postoperative and follow-up images were evaluated by two independent authors. If any disagreements arose, they were resolved by discussion, and the senior author made the final determination.

### Statistical analysis

SPSS version 13.0 was used for all statistical analyses. The paired sample *t*-test was utilized to compare ODI scores, JOA scores, DHI and ROM. *p*-values of less than 0.05 were considered to indicate statistical significance.

## Results

A total of 38 consecutive patients were included in this study. There were two dropouts: one from unrelated death due to a cerebral infarction and the other with incomplete data due to a change of residence two months after operation. Five patients failed to complete all the clinical and radiographic evaluations at all time intervals, and hence were excluded. The results of the examination are based on the analysis of data obtained from 31 patients. Baseline characteristics are showed in Table 1. Stabilizations were performed at L1/2 in one patient, L2/3 in two patients, L3/4 in four patients, L4/5 in ten patients, L5/S1 in six patients, L3-L5 in six patients, L5-S1 in two patients. There were 11 cases of lumbar intervertebral disc herniation, 12 of degenerative lumbar spinal stenosis, 6 of lumbar instability syndrome, and 2 of recurrent lumbar disc herniation. Nerve root decompression or limited laminectomy was performed in 26 patients, out of whom in 13 it was combined with discectomy, whereas in the 5 other cases simple fixation without decompression was conducted.

**Table 1:** Patients characteristics

Number	31
Mean age (range), years	56.3 (35–75)
Gender (male/female)	12/19
Follow-up (range), months	23.8 (21–26)
Mean operative time (range), mins	97.4 (50–180)
Mean blood loss (range), mls	234 (100–800)

Two perioperative complications occurred: one case of dural tear that was sutured, and one of fat liquefaction of incision. No revision surgery or removal of implants was reported in any of the patients.

### Clinical outcome

**Fig. 2:**
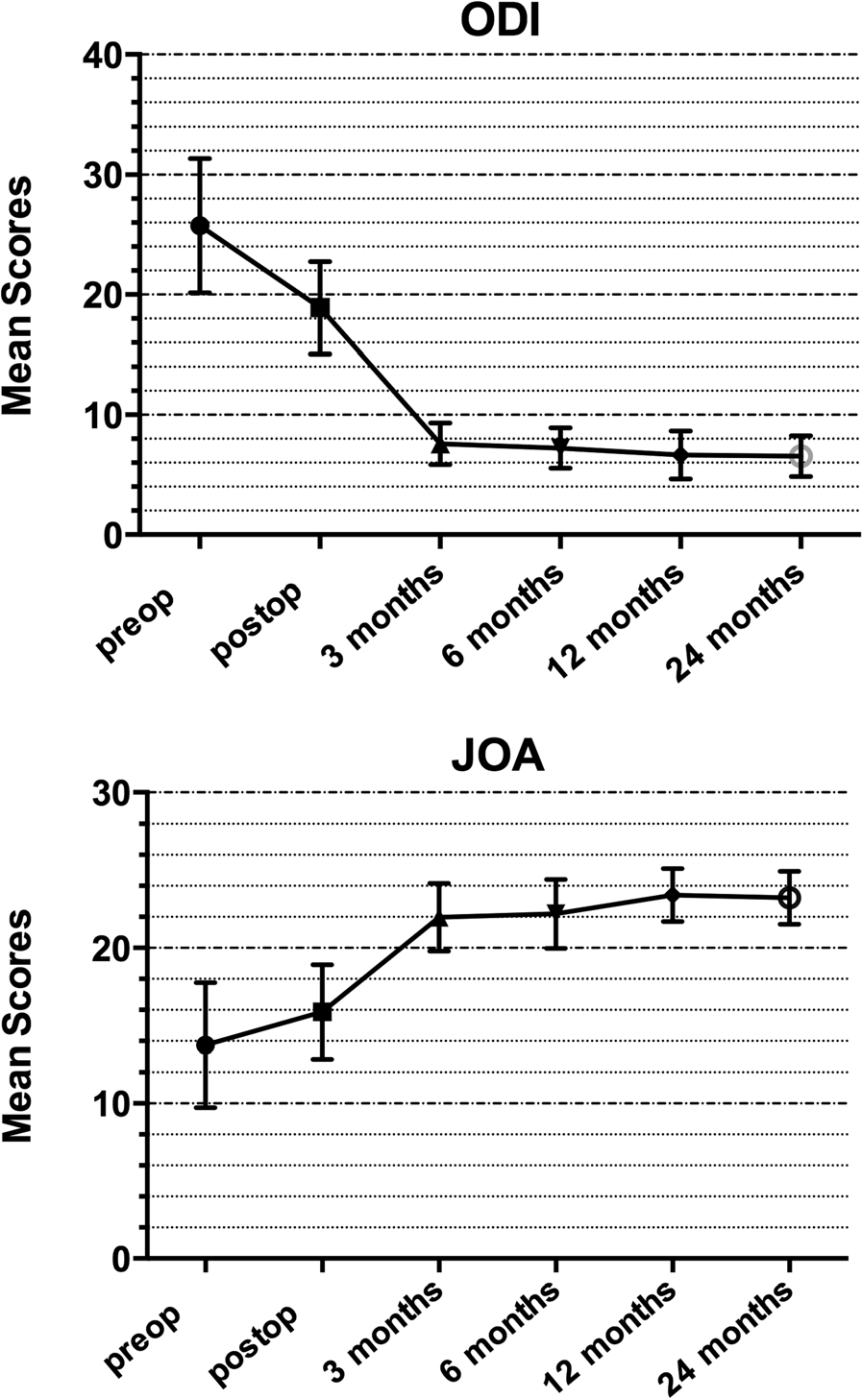
Clinical results of non-fusion surgery using PEEK rod systems measured by *ODI* (Oswestry disability index) scores and *JOA* (Japanese orthopaedics association) scores

Mean JOA scores improved from 13.7 (range 7–19) to 23.2 (range 18–26) at the final follow-up (*p* <0.05). A decline was noticed in mean ODI scores from 25.7 (range 18–35) to 6.5 (range 4–11) at the final follow-up (*p* <0.05) (Fig. 2).

### Radiographic outcome

**Fig. 3:**
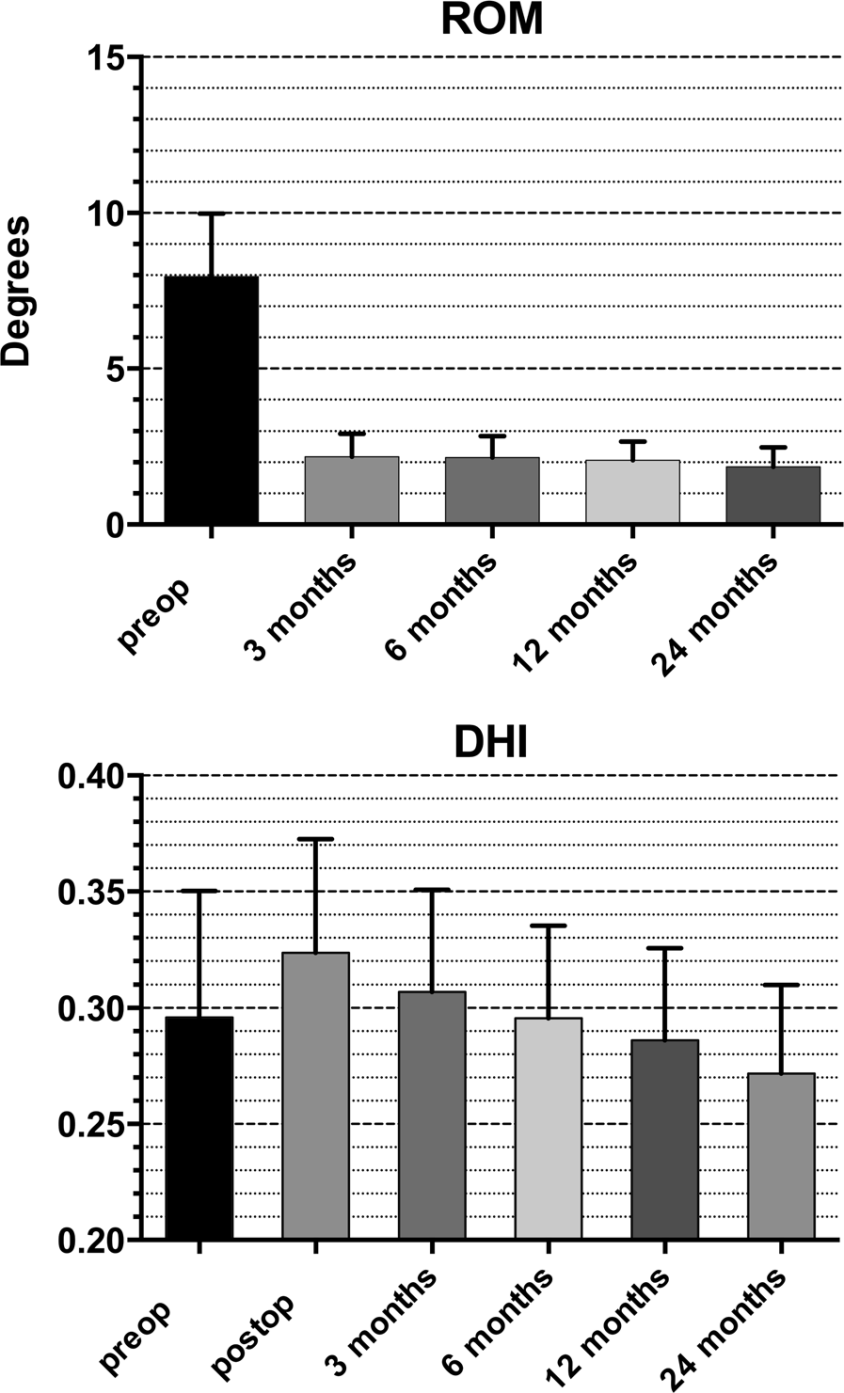
Radiographic outcomes of *ROM* (range of motion) and *DHI* (disc height index). The *ROM* was measured by flection and extension fluoroscopy on dynamic radiographs

The mean DHI increased from a preoperative value of 0.30 (range 0.17–0.38) to a mean postoperative value of 0.32 (range 0.19–0.41), and then a decline was observed to 0.27 (range 0.19–0.34) at the final follow-up (Fig. 3). No statistical difference was detected between the preoperative DHI values and those established at the final follow-up (*p* >0.05).

The mean ROM on flexion–extension views at the stabilized levels was 8.8° (range 4.9–17.2) before surgery and declined to 2.1° (range 0.9–3.9) at 3 months after surgery and 1.8° (range: 0.9–3.2) at the final follow-up (*p* <0.05) (Fig. 3).

**Fig. 4:**
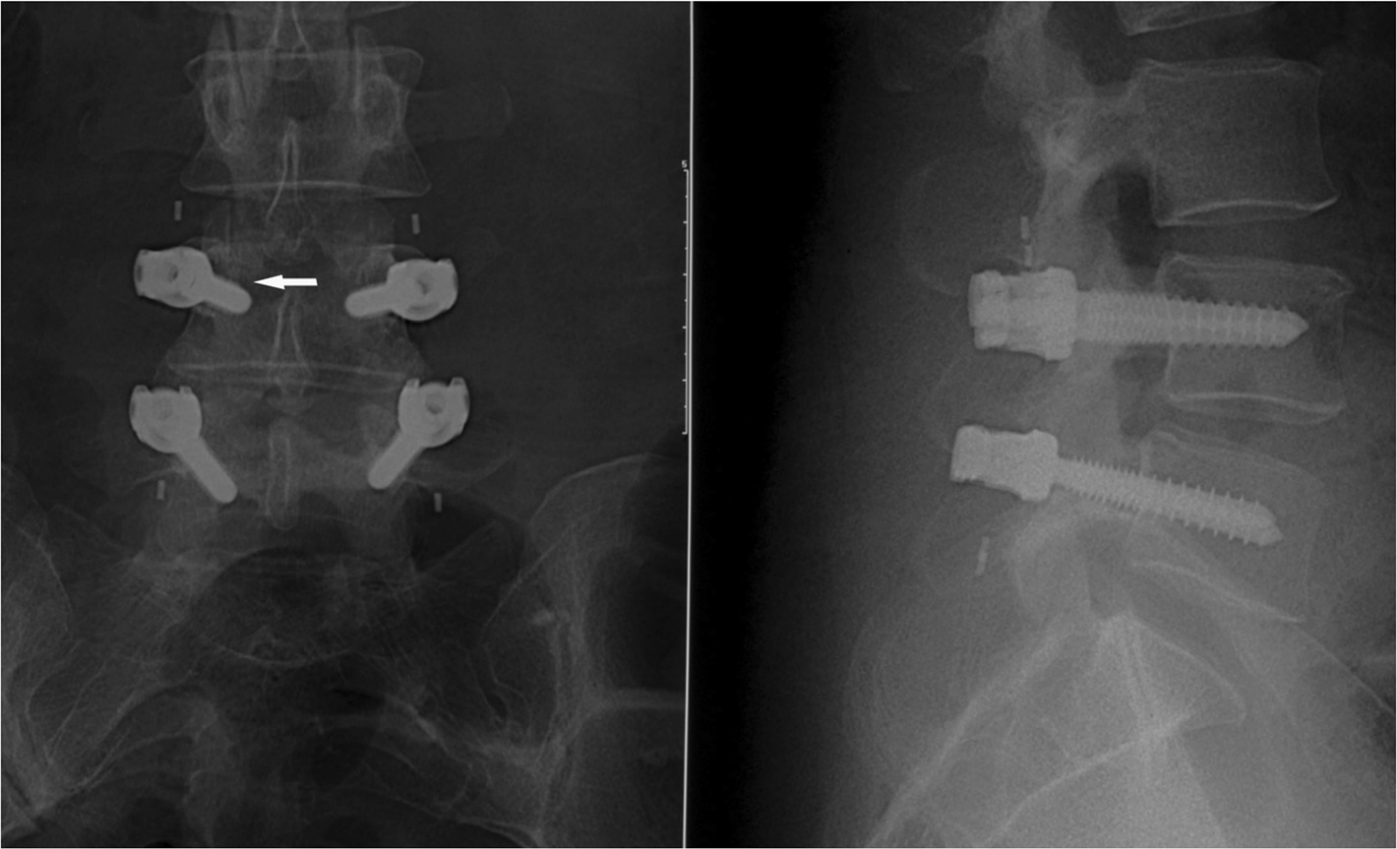
X-ray radiographs showed halo sign (*white arrow*) around right L4 pedicle screw, which indicated screw loosening

Anterior-posterior and lateral lumbar radiographs showed screw loosening in one patient without symptoms (Fig. 4). Three-dimensional reconstruction of CT scan images demonstrated no rod breakage at the final follow-up.

## Discussion

In this study, 38 consecutive cases of non-fusion surgery using PEEK rod systems for lumbar degenerative diseases were investigated. Seven patients failed to provide the complete data. The results of the 31 cases demonstrated favorable outcomes. JOA scores and ODI scores improved substantially postoperatively. No serious complications occurred during the entire follow-up period.

The clinical effects of PEEK rod systems as a non-fusion device in this study, compared to those of Dynesys and Isobar TTL, were similar to the ones reported previously. Lee et al. [21] found that the mean visual analogue scale (VAS) scores of 20 consecutive patients who underwent decompression with Dynesys system decreased from 8.5 to 2.2 and mean ODI declined from 79.58 to 22.17 %. The authors concluded that Dynesys could provide clinical improvements in patients with degenerative spinal diseases. In a multicenter trial, Stoll et al. [17] established that the mean VAS of back pain and leg pain of 83 patients undergoing Dynesys decreased from 7.4 to 3.1 and from 6.9 to 2.4, respectively. The mean ODI score declined from 55.4 to 22.9 %. The Dynesys system was concluded to be a safe and effective alternative for unstable lumbar conditions.

Excellent early clinical results from the application of the fusion procedure by PEEK rod systems were obtained in several clinical studies [13, 14, 16]. De Iure et al. [14] retrospectively reviewed 30 cases in which posterior fusion was supported by PEEK rod systems and found that the clinical results were satisfactory at an average length of the follow-up period of 18 months. Athanasakopoulos et al. [13] described a clinical series of 52 patients who underwent posterior spinal fusion using the PEEK rod system between 2007 and 2010. The mean follow-up duration was 3 years. ODI scores improved from 38 preoperatively to 15 at 1 year postoperatively. Mean low back and leg VAS scores improved from 8 and 9 points preoperatively to 2 points at 1 year postoperatively. Qi et al. [16] carried out a prospective control study to compare the clinical outcomes of posterior lumbar interbody fusion by PEEK rods versus titanium rods utilization. Postoperative VAS and JOA scores improved significantly in both the PEEK rods group and the titanium rods group and no statistical difference was detected between the groups in the improvement of clinical outcomes.

Less rigid pedicle-based stabilization was advocated to preserve a certain degree of motion. In the current study, the use of PEEK rod systems caused a decrease in ROM values from 8.8° preoperatively to 1.8 postoperatively, as determined at the final follow-up. The findings of the current study were in line with the results of cadaveric testing conducted by Ponnappan et al. [11], which demonstrated that PEEK rod systems can significantly reduce the ROM of a destabilized segment from a mean value of 8.49° to 2.09° in flexion–extension.

In fact, according to the related literature and data available, the use of PEEK rod systems and Dynesys surgical technique might have a similar impact on ROM. Cadaveric testing conducted by Schulte et al. [22] showed that ROM in flexion–extension was reduced from the mean 8.1° to 2.0° after Dynesys implantation. A recent finite element analysis also demonstrated similar results. A lumbar functional unit combined with pedicle-based dynamic stabilization were constructed to simulate postoperative changes using Dynesys, PEEK rod systems, N-Flex, and traditional titanium rod. The data obtained also displayed similar changes in ROM after the application of PEEK rod and Dynesys systems [5]. Undoubtedly, PEEK rods and Dynesys implants have different designs and different elasticity moduli. However, it has been revealed that stabilizations only of the modulus with a very low degree of stiffness influenced ROM levels markedly [22, 23]. Specifically, the relationship between modulus and ROM do not exhibit a linear dependence. The ROM merely varies significantly when the modulus is reduced below a specific threshold value and the moduli of PEEK rods and Dynesys implants are above the threshold values. Thus, theoretically, the different modulus of PEEK rod systems and Dynesys implants may not lead to distinct ROM in vivo and it is feasible that PEEK rods could serve as non-fusion fixation since the Dynesys system has also been used in the fusion procedure [24].

At the stabilized levels, DHI at stabilized levels increased slightly postoperatively, but gradually declined and dropped below preoperative levels at the 2-year follow-up. In fact, pedicle-based dynamic fixation systems are not suitable for restoring disc height. Cienciala conducted a retrospective study in 102 patients with Dynesys instrument and found that the disc height reduction in the anterior segment was by up to 0.7 mm [25]. In a Beastall’s study [26], the postoperative images of 24 patients treated by the Dynesys showed that the mean anterior disc height was reduced by 0.7 mm and the mean posterior disc height was decreased by 0.3 mm after the insertion of the Dynesys implants. Similar results were obtained for Isobar TTL. As reported separately by Li et al. [27] for 37 consecutive patients and Fu et al. [28] for 36 patients, immediately after the operation, DHI increased by 20 and 5 %, respectively. However, in Li’s study DHI values declined significantly to 11 % at the 2-year follow-up, whereas in Fu’s study DHI decreased by 10 %, as compared with the preoperative values at the 2-year follow-up. In contrast, DHI improved more significantly in patients who received interbody fusion [29, 30]. Therefore, the lack of support by a cage in the anterior column contributes to unsatisfying DHI in patients with pedicle-based dynamic stabilization. In addition, the techniques of distraction may account for the differences. To prevent scratching, direct distraction was not allowed in our trial. Retrieval analysis demonstrated that scratching on the PEEK rods surface might result in PEEK debris and cause inflammation [31]. Thus, during the operation, alloy rods were first placed on both sides firstly and then distraction was carried out. Further, the PEEK rods were positioned respectively in the place of the alloy rods on both sides. Due to the existing tension between segments, a part of the achieved distraction might have been lost in the process of replacement.

Another argument against the non-fusion procedure is the possible implant failure [17, 32]. Three-dimensional reconstruction of CT scans revealed no rod breakage in our study. Actually, to our knowledge no PEEK rod breakage has been reported until now [13–16]. In one case, asymptomatic screw loosening was manifested at the last follow-up. Based on the finding of some previous studies that demonstrated screw loosening was the most common complication for pedicle-based dynamic stabilization [17, 33], it seems that PEEK rod systems have superior implant safety. PEEK rod systems are believed to potentially lower the possibility of implant failure, such as screw loosening, and have been evidenced to optimize load sharing and reduced stress at the bone-screw interface, as determined by cadaveric testing [11] and finite element studies [10]. Nonetheless, the PEEK rod systems used in the current experimental series employed a dual-lead pedicle screws which have more threads at the tail. These newly designed screws need greater insertion torque and may have a superior screw-to-bone purchase within the pedicle, which could increase pullout strength and facilitate enhanced outcome.

Several limitations of the current study should be noted in the current study. First, despite the statistical improvement in ODI and JOA scores, it was not confirmed that this effect was caused by the decompression procedure or the dorsal stabilization. Second, the sample of patients in the current examination demonstrated high variance in disease pattern, instrumented levels and age, which might have generated bias. Third, the small sample size of the present investigation was relatively small.

## Conclusion

Despite these limitations, the obtained preliminary results indicated that the non-fusion procedure of PEEK rod systems might be a viable alternative for treatment of lumbar degenerative disease. The implant failure rate was lower compared to those reported in the literature. A more effective distraction technique needs to be explored for disc height restoration.

## Abbreviations

PEEK: polyetheretherketone
BMI: body mass index
DEXA: a dual energy X-ray absorptiometry
ODI: Oswestry Disability Index
JOA: Japanese Orthopaedics Association
DHI: disc height index
ROM: range of motion
